# Association Between Consumption of Sugar-Sweetened Beverages and Sociodemographic Characteristics Among Mississippi Adults

**DOI:** 10.5888/pcd14.170268

**Published:** 2017-12-21

**Authors:** Vincent L. Mendy, Rodolfo Vargas, Marinelle Payton, Gerri Cannon-Smith

**Affiliations:** 1 Department of Epidemiology and Biostatistics, School of Public Health, Jackson State University, Jackson, Mississippi; 2 Office of Health Data and Research, Mississippi State Department of Health, Jackson, Mississippi; 3 Institute of Epidemiology and Health Services Research, Center of Excellence in Minority, Health and Health Disparities, School of Public Health, Jackson State University, Jackson, Mississippi

## Abstract

**Introduction:**

The consumption of sugar-sweetened beverages (SSBs) is linked to excessive weight gain, diabetes, and risk of cardiovascular disease. We examined the association between SSB consumption and sociodemographic characteristics among Mississippi adults.

**Methods:**

We used data from the 2012 Mississippi Behavioral Risk Factor Surveillance System, which collected information on SSB consumption from 7,485 respondents. We used logistic regression models to calculate adjusted prevalence ratios (APRs) and 95% confidence intervals (CIs) for characteristics associated with SSB consumption.

**Results:**

In 2012, 40.8% of Mississippi adults reported consuming at least one SSB daily. The likelihood of consuming SSBs at least once daily among respondents aged 18 to 34 years was 2.81 times higher (APR, 2.81; 95% CI, 2.49–3.18) than among those aged 65 years or older. The prevalence among men was 20% higher (APR, 1.20; 95% CI, 1.11–1.30) than among women and 23% higher (APR, 1.23; 95% CI, 1.13–1.35) among black respondents than among white respondents. The prevalence among respondents with less than a high school education was 25% higher (APR, 1.25; 95% CI, 1.11–1.41) than among those who with more than a high school education and 33% higher (APR, 1.33; 95% CI, 1.16–1.52) among those with an annual household income of less than $20,000 than among those with an income of $50,000 or more.

**Conclusion:**

Among Mississippi adults, age, sex, race, education level, and income are associated with an increased likelihood of SSB consumption. Findings highlight the need for policies and interventions to address SSB consumption and promote alternatives to SSBs among Mississippians.

## Introduction

Sugar-sweetened beverages (SSBs), including regular soda, sports drinks, fruit drinks, energy drinks, and caloric-sweetened water are a major source of added sugars in US diets (1,2). On average, US adults consume 145 kcal daily from SSBs; 6.5% of their daily energy intake is from SSBs (3). Consumption of SSBs is associated with excessive weight gain, obesity, diabetes, hypertension, and cardiovascular disease (4–6). In Mississippi in 2012, heart disease, diabetes, and hypertension were the first, seventh, and twelfth leading causes of death, respectively, together accounting for 30% of all deaths (7). In 2012, Mississippi had the second highest adult obesity rate (34.6%) in the nation, with more than two-thirds of adults (68.9%) being overweight or obese (8). A study among Mississippi adults found that the prevalence of obesity increased 2.9% annually from 2001 to 2010 (9).

Previous cross-sectional studies found associations between the consumption of SSBs and sociodemographic characteristics at the national and state levels and in primary care settings (10–13). Information on how SSB consumption is associated with sociodemographic characteristics among Mississippi adults is limited. The objective of this study was to describe SSB consumption among Mississippi adults and examine the associations between SSB consumption and sociodemographic characteristics.

## Methods

We analyzed data from the 2012 Mississippi Behavioral Risk Factor Surveillance System (BRFSS), which included an optional module that assessed SSB consumption. The BRFSS is a state-based, random–digit–dialed telephone survey of the US noninstitutionalized civilian population aged 18 years or older. The survey is conducted in all 50 states, the District of Columbia, and 3 US territories (Puerto Rico, Guam, and the US Virgin Islands). Data from the BRFSS provide reliable and valid assessments of health risk factors (14). The BRFSS was approved by human research review boards from departments of health in each state. Detailed information about BRFSS is available (www.cdc.gov/brfss/). Analyses were restricted to respondents who self-identified as black or white (N = 7,485); these racial groups accounted for 96.6% of the Mississippi population in the 2010 Census (7). This study was deemed exempt by the Mississippi State Department of Health institutional review board.

Respondents were asked 2 questions about their consumption of SSBs: 1) “During the past 30 days, how often did you drink regular soda or pop that contains sugar? Do not include diet soda or diet pop” and 2) “During the past 30 days, how often did you drink sweetened fruit drinks, such as Kool-Aid, cranberry juice cocktail, and lemonade? Include fruit drinks you made at home and added sugar to.” For each question, respondents reported the number of times per day, per week, or per month they consumed these drinks. Weekly or monthly consumption was converted to daily consumption (dividing weekly consumption by 7 and monthly consumption by 30). To calculate the overall prevalence of SSB consumption, the consumption of regular soda or pop and the consumption of sweetened fruit drinks were summed (12,15). SSB consumption was defined as the proportion of those who consumed SSBs at least once daily (10,12,13).

Sociodemographic variables were age (18–34 y, 35–49 y, 50–64 y, or ≥65 y), sex, race (black or white), education level (<high school graduate, high school graduate or equivalent, or >high school graduate), and annual household income (<$20,000; $20,000–$34,999; $35,000–$49,999; ≥$50,000; or Don’t know/refused).

We used χ^2^ tests to assess the associations between SSB consumption and sociodemographic characteristics. A logistic regression model was then used to estimate adjusted prevalence ratios (APRs) and 95% confidence intervals (CIs) for these associations, using predicted marginals (16). The logistic model included age, sex, race, education, and annual household income. SAS version 9.4 (SAS Institute, Inc) was used to perform all statistical analyses, accounting for the complex sample design. Results were significant at *P* <.05.

## Results

Mean age of respondents was 57.2 years. By age, the greatest percentage (30.8%) of respondents were aged 18 to 34 years. More than one-third (37.3%) were black, more than half (52.4%) were women, and more than one-quarter (27.2%) had an annual household income of less than $20,000 (Table 1). Two of every 5 adults (40.8%) consumed at least one SSB per day. Daily consumption of SSBs was most prevalent among adults aged 18 to 34 years, men, black respondents, respondents with less than a high school education, and those with an annual household income of less than $20,000 (Table 2).

**Table 1:** Sociodemographic Characteristics of Mississippi Adults (N = 7,485), 2012 Behavioral Risk Factor Surveillance System^a^

Characteristic	No. (%)^a^^,^ b	95% Confidence Interval
**Age, y**		
18–34	960 (30.8)	29.0–32.5
35–49	1,323 (24.6)	23.2–26.1
50–64	2,426 (25.8)	24.6–27.1
≥65	2,776 (18.8)	17.9–19.6
**Race**		
Black	2,569 (37.3)	35.7–38.9
White	4,871 (62.7)	61.1–64.3
**Sex**		
Male	2,621 (47.6)	45.9–49.2
Female	4,864 (52.4)	50.8–54.1
**Education level**		
<High school graduate	1,276 (19.7)	18.3–21.2
High school graduate or equivalent	2,445 (30.5)	29.0–31.9
>High school graduate	3,750 (49.8)	48.1–51.5
**Annual household income, $**		
<20,000	2,102 (27.2)	25.7–28.7
20,000–34,999	1,557 (20.7)	19.4–22.1
35,000–49,999	783 (11.2)	10.1–12.2
≥50,000	1,835 (26.0)	24.6–27.4
Don’t know/refused	1,208 (15.0)	13.8–16.2

a Some values for n may not sum to total because of missing data.

b Unweighted number and weighted percentage.

**Table 2:** Association Between Sugar-Sweetened Beverage Consumption and Sociodemographic Characteristics Among Mississippi Adults, 2012 Behavioral Risk Factor Surveillance System

Characteristic	Sugar-Sweetened Beverage^a^ Consumption			*P* Value^c^
	None, %^b^ (95% CI)	<1 Time/Day, %^b^ (95% CI)	≥1 Time/Day, %^b^ (95% CI)	
**Overall**	24.0 (22.7–25.3)	35.2 (33.6–36.8)	40.8 (39.1–42.5)	NA
**Age, y**				
18–34	10.4 (7.9–13.0)	29.9 (26.3–33.4)	59.7 (55.8–63.6)	<.001
35–49	20.0 (17.3–22.6)	36.6 (33.3–39.9)	43.4 (39.9–46.9)	
50–64	30.8 (28.4–33.2)	38.1 (35.5–40.6)	31.2 (28.6–33.8)	
≥65	41.7 (39.4–44.1)	37.9 (35.6–40.2)	20.4 (18.4–22.4)	
**Sex**				
Male	21.3 (19.2–23.3)	33.8 (31.3–36.3)	44.9 (42.2–47.7)	<.001
Female	26.5 (24.8–28.2)	36.4 (34.4–38.4)	37.1 (35.0–39.2)	
**Race**				
Black	12.9 (11.0–14.7)	35.3 (32.6–38.1)	51.8 (48.8–54.7)	<.001
White	30.7 (28.9–32.4)	35.0 (33.0–36.9)	34.4 (32.3–36.4)	
**Education level**				
<High school graduate	21.3 (18.2–24.4)	31.0 (27.1–34.8)	47.7 (43.4–52.0)	<.001
High school graduate or equivalent	24.0 (21.7–26.4)	31.7 (29.1–34.2)	44.3 (41.3–47.3)	
>High school graduate	25.1 (23.3–27.0)	39.0 (36.7–41.2)	35.9 (33.6–38.2)	
**Annual household income, $**				
<20,000	18.0 (15.8–20.2)	32.4 (29.4–35.4)	49.6 (46.2–53.0)	<.001
20,000–34,999	23.3 (20.4–26.1)	34.2 (30.7–37.7)	42.5 (38.7–46.3)	
35,000–49,999	23.9 (19.6–28.2)	36.3 (31.7–41.0)	39.7 (34.7–44.8)	
≥50,000	29.5 (26.8–32.2)	40.2 (37.1–43.3)	30.4 (27.3–33.5)	
Don’t know/refused	26.8 (23.3–30.4)	32.0 (28.1–35.9)	41.2 (36.7–45.7)	

Abbreviations: CI, confidence interval; NA, not applicable.

a Sugar-sweetened beverages include fruit drinks and regular soda.

b Weighted percentage.

c Determined by χ^2^ test for ≥1 time/day.

Compared with respondents aged 65 years or older, the likelihood of consumption of SSBs at least once daily among respondents aged 18 to 34 years was 2.81 times higher (*P* < .001), among those aged 35 to 49 years was 2.18 times higher (*P* < .001), and among those aged 50 to 64 years was 54% higher (*P* < .001) (Table 3). The likelihood of consumption of SSBs at least once daily among men was 20% higher (*P* < .001) than among women and among black respondents was 23% higher (*P* < .001) than among white respondents. The likelihood of consumption of SSBs at least once daily among respondents with less than high school education was 25% higher (*P* < .001) and among those with high school diploma or equivalent was 16% higher (*P* = .001) than among those with more than a high school education. Compared with respondents with an annual household income of $50,000 or more, the likelihood of consumption of SSB at least once daily among those with an annual household income of less than $20,000 was 33% higher (*P* < .001), among those with an annual household income of $20,000 to $34,999 was 22% higher (*P* = .003), and among those with an annual household income of $35,000 to $49,999 was 19% higher (*P* = .03).

**Table 3:** Adjusted Prevalence Ratios of Consumption of Sugar-Sweetened Beverages at Least Once Per Day Among Mississippi Adults, 2012 Behavioral Risk Factor Surveillance System

Characteristic	Adjusted Prevalence Ratio^a^ (95% Confidence Interval)	*P* Value
**Age, y**		
18–34	2.81 (2.49–3.18)	<.001
35–49	2.18 (1.91–2.48)	<.001
50–64	1.54 (1.35–1.76)	<.001
≥65	1 [Reference]	
**Sex**		
Male	1.20 (1.11–1.30)	<.001
Female	1 [Reference]	
**Race**		
Black	1.23 (1.13–1.35)	<.001
White	1 [Reference]	
**Education level**		
<High school graduate	1.25 (1.11–1.41)	<.001
High school graduate or equivalent	1.16 (1.06–1.29)	.001
>High school graduate	1 [Reference]	
**Annual household income, $**		
<20,000	1.33 (1.16–1.52)	<.001
20,000–34,999	1.22 (1.07–1.40)	.003
35,000–49,999	1.19 (1.02–1.38)	.03
≥50,000	1 [Reference]	
Don’t know/refused	1.20 (1.03–1.39)	.02

a Adjusted for age, sex, race, education level, and annual household income.

## Discussion

To our knowledge, our study is the first to examine the associations between SSB consumption and sociodemographic characteristics among Mississippi adults. The results demonstrated that in 2012, an estimated 2 of every 5 Mississippi adults reported consuming at least one SSB per day. Consumption of SSBs at least once per day significantly differed by sociodemographic factors among Mississippi adults. Study findings also indicated significant associations between daily SSB consumption and 5 sociodemographic factors — age, race, sex, education level, and annual household income — among adult Mississippians.

The finding that increased likelihood of consuming at least one SSB per day among younger adults, men, black respondents, those with less than a high school education, and those with an annual household income of less than $20,000 mirror the results of previous studies (12,17,18). A study in the rural Lower Mississippi Delta found that health literacy significantly predicted SSB consumption (19); men had significantly lower health literacy scores and higher consumption of SSBs than women, while black participants had significantly lower health literacy skills and higher consumption of SSB than white participants (19). Therefore, low levels of health literacy may be partially responsible for the positive association between SSB consumption among men and black respondents in our study. Health literacy includes oral literacy, print literacy, media literacy, numeracy, and conceptual knowledge (19).

The Centers for Disease Control and Prevention (CDC) outlined strategies such as ensuring ready access to potable drinking water, decreasing the relative cost of more healthful beverages through differential pricing of SSBs, and expanding the knowledge and skills of medical care providers to include screening for high SSB consumption and counseling on reducing SSB consumption (20).

Taxes on soda are promoted as an effective strategy for reducing SSB consumption. For example, Berkeley, California, implemented taxes on SSBs, a strategy that reduced SSB consumption in low-income neighborhoods (21). In addition, researchers found that point-of-purchase interventions decreased sales of SSBs (22). A systematic review of the price elasticity of demand for major food categories demonstrated that a 10% increase in soft drink prices could reduce consumption by 8% to 10% (23). Wang and colleagues showed that a penny-per-ounce tax on SSBs could reduce consumption by 15% among adults and prevent an estimated 2.4 million diabetes person-years, 95,000 coronary heart events, 8,000 strokes, and 26,000 premature deaths between 2010 and 2020 (24). Cardiovascular disease (CVD) is the leading cause of death in Mississippi; in 2012 the state’s CVD death rate was 1.3 times the national rate (25). Evidence indicates that reducing SSB consumption leads to reduced prevalence of obesity and obesity-related diseases (26). In 2012, an estimated 738,512 (34.6%) Mississippi adults were obese (8). Mississippi taxes soda at the same rate as other foods and beverages and could consider implementing a tax on SSBs.

To be successful, interventions that reduce SSB consumption should include collaborative preventive health strategies (public–private partnerships), educational strategies (health/health literacy), economic strategies, and counter-marketing strategies.

A strength of this study was using a representative sample of the adult Mississippi population. However, our study has limitations. First, the BRFSS data on SSB consumption were self-reported, so they are subject to recall and social desirability bias (27). Second, the optional module for assessing SSB consumption used in 2012 in Mississippi included only 2 types of SSBs (regular soda and fruit drinks); data on other SSBs, such as sports drinks, iced tea, and energy drinks were not available. Third, the structure of questions on SSB consumption prevented us from determining the specific amount of SSBs consumed (15). Finally, the associations were cross-sectional and do not permit causal inferences.

Prevention initiatives such as educational campaigns and policies aimed at reducing SSB consumption are warranted in Mississippi, particularly because the state has high rates of obesity, diabetes, and CVD, all of which are linked to SSB consumption (4,24,26). Focused educational campaigns and policies should target Mississippians who have an increased likelihood of consuming SSBs, such as young adults, men, black individuals, people with less than a high school education, and those with an annual household income of less than $20,000. For example, interventions that aim to improve the health literacy on SSB consumption and its associated health consequences could help people in these groups. However, the uneven distribution of literacy levels, the intransigence of poverty, adherence to current policies, and the adequacy and effectiveness of initiatives for economic development could impede public health efforts to reduce SSB consumption in the state.

State Public Health Actions to Prevent and Control Diabetes, Heart Disease, Obesity, and Associated Risk Factors and Promote School Health (DP13–1305) (28) through partnerships with national and local stakeholders must include promotion and prevention campaigns that focus on a reduction of SSB consumption and must target people who are most likely to consume SSBs. Reducing the consumption of SSBs could help reduce rates of hypertension, obesity, and diabetes in Mississippi.
